# A comparative study of anti-aging properties and mechanism: resveratrol and caloric restriction

**DOI:** 10.18632/oncotarget.20084

**Published:** 2017-08-09

**Authors:** Juan Li, Chun-Xia Zhang, Yi-Mei Liu, Ke-Li Chen, Gang Chen

**Affiliations:** ^1^ Key Laboratory of Ministry of Education on Traditional Chinese Medicine Resource and Compound Prescription, Hubei University of Chinese Medicine, Wuhan 430065, Hubei, China; ^2^ Hubei College of Chinese Medicine, Jingzhou 434020, Hubei, China

**Keywords:** resveratrol, caloric restriction, SIRT1, aging

## Abstract

Resveratrol and caloric restriction (CR) are the powerful therapeutic options for anti-aging. Here, their comparative effect on longevity-associated gene silencing information regulator (SIRT1) were evaluated *in vitro* and *in vivo*. IMR-90 cells treated with 2,2’-azobis (2-amidinopropane) dihydrochloride (AAPH) were applied to establish a cellular senescence model, and rats treated with D-galactose (D-gal) were used as an aging animal model. Resveratrol and CR exhibited similar anti-aging activities, evidenced by inhibiting senescence and apoptosis, and restoring cognitive impairment and oxidative damage. Moreover, they could up-regulate telomerase (TE) activity, increase expressions of SIRT1, forkhead box 3a (Foxo3a), active regulator of SIRT1 (AROS) and Hu antigen R (HuR ), but decrease p53 and deleted in breast cancer 1 (DBC1) levels. However, 10 μM resveratrol *in vitro* and the high dose group *in vivo* showed relatively stronger activities of anti-aging and stimulating SIRT1 level than CR. In conclusion, resveratrol and CR showed similar anti-aging activities on SIRT1 signaling, implicating the potential of resveratrol as a CR mimetic.

## INTRODUCTION

Improvements in health care have helped raise human life expectancy in recent decades, and the elderly population is thus increasing significantly. Unfortunately this still means the increasing periods of poor health or disability. Currently, a variety of aging-related diseases are emerging as the greatest health threats in most developed countries. Although it is not yet possible to modify our genetic background, various anti-aging strategies are currently emerged as healthy lifestyles and therapeutic interventions, aiming to reduce the incidence of risk factors of poor health [1].

CR, decreased calorie intake without malnutrition, is one of the most robust interventions that increase lifespan in model organisms from yeast to primates [2]. It could protect against the deterioration of biological functions, reducing the incidence and delaying the onset of multiple age-related diseases. The mechanism by which CR prolongs lifespan involves retardation of growth, reduction of body fat, delaying neuroendocrine or immunologic changes, increase in DNA repair capacities, altered gene expression, enhanced apoptosis, reduction of body tempetature and depression of metabolic rate, and amelioration oxidative stress [3]. The anti-aging effect of CR has been strongly associated with an increased level and activation of members of the sirtuin family, and also related to other molecular signaling pathways, including peroxisome proliferator activated receptor G coactivator-1α (PGC-1α), adenosine monophosphate activated protein kinase (AMPK), insulin/insulin growth factor-1, and target of rapamycin [1, 4]. However, most people would not comply with such a rigorous dietary program, particularly in the long term. Therefore, recent research is increasingly aimed at determining the feasibility and efficacy of natural and/or pharmacological CR mimetic molecules/ treatments without decreasing food consumption [5].

Resveratrol is a plant polyphenol commonly found in various plants, including the skin of grapes, berries, and peanuts. Interestingly, incorporation of resveratrol into dietary supplements or foods may be a powerful therapeutic option for anti-aging, and several reports indicate that resveratrol treatment produces beneficial effects similar to those of CR [6], implicating the potential of resveratrol as a CR mimetic [4]. Recently, it has been demonstrated that resveratrol extends the lifespan through significantly increasing SIRT1 activity, resulting in the increase of SIRT1 affinity for both NAD+ and the acetylated substrate [7, 8], which is also responsible for the longevity caused by CR. Although resveratrol and CR have been widely studied for their potential health benefits, little is known about their comparative effects. In this study we compared the anti-aging effect of resveratrol and CR *in vivo* and *in vitro* through detecting SIRT1 pathway.

## RESULTS

### Resveratrol and CR decreased senescence-associated β-galactosidase (SA β-gal) staining in AAPH-treated IMR-90 cells

**Figure 1:**
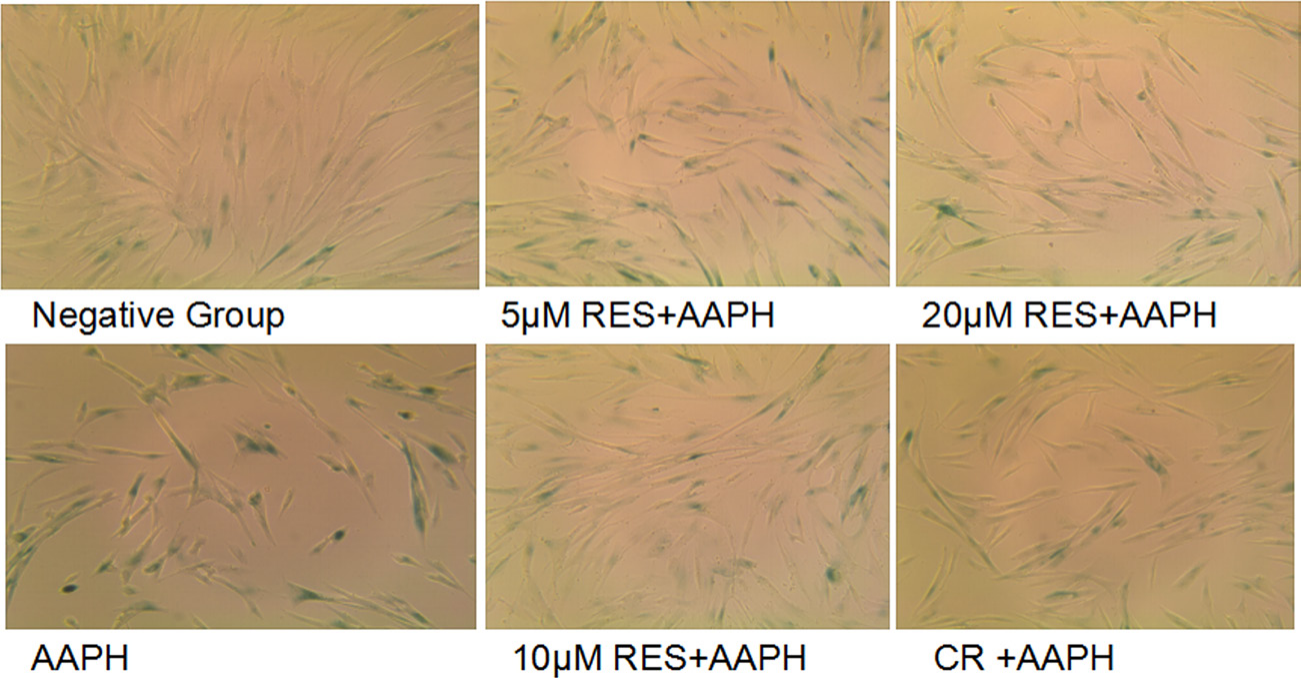
Resveratrol and caloric restriction decreases SA-β-gal staining in AAPH-treated IMR-90 cells AAPH induced IMR-90 cells were treated with caloric restriction (CR) or resveratrol (RES) at the indicated concentration for 2 days. The magnification was 20×.

Taking advantage of an AAPH-induced senescence model of IMR-90 cells, we tested the possible effect on SA-β-gal staining. As shown in Figure 1, the AAPH-treated cells displayed more widely and strongly positive staining than did in control cells, and the positive staining in control cells was relatively scarce. Resveratrol (5 μM, 10 μM and 20 μM) or CR treatment was found to afford markedly protections in AAPH-induced senescence. Moreover, compared the effect of resveratrol with CR, cells treated wiht 10 μM resveratrol displayed relatively less often SA-β-Gal positive staining than CR treatment. Thus, these results suggested that 10 μM resveratrol more efficiently inhibited AAPH-induced senescence than CR.

### Scanning electron microscopy analysis of IMR-90 cells

**Figure 2:**
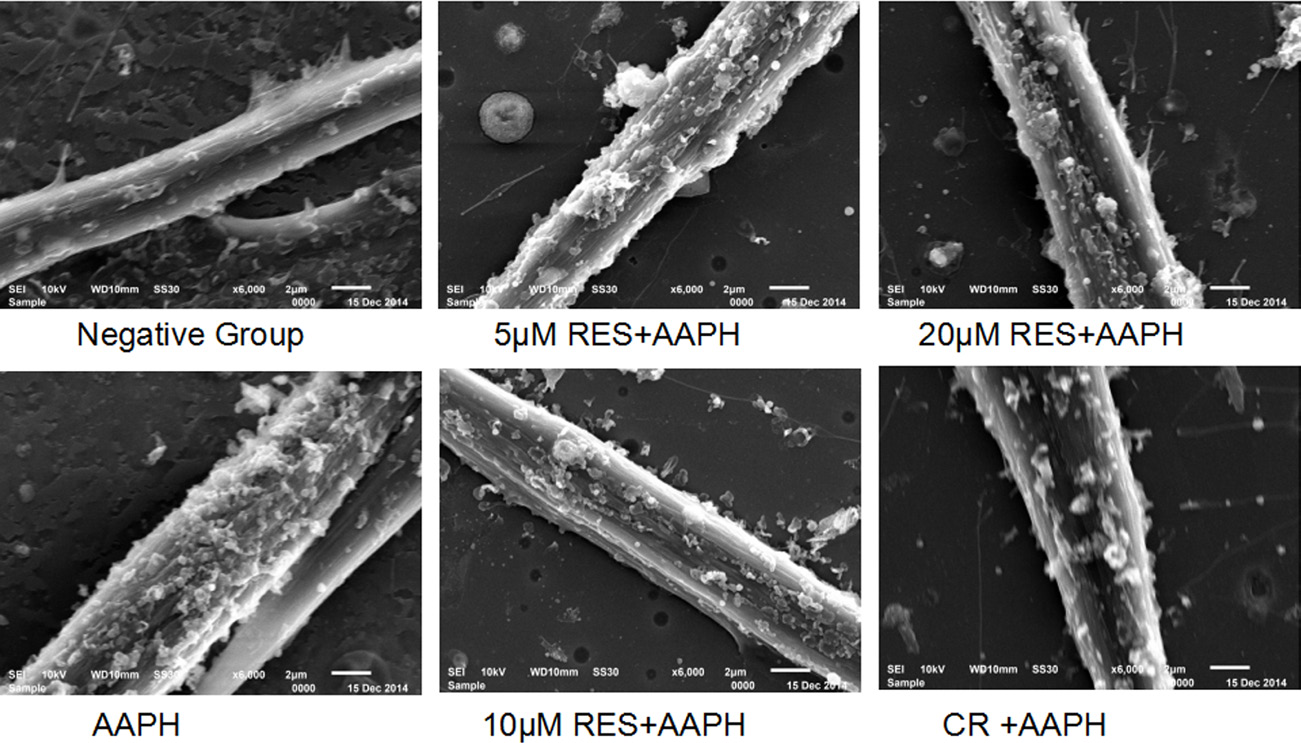
Scanning electron microscopy analysis of IMR-90 cells AAPH induced IMR-90 cells were treated with caloric restriction (CR) or resveratrol (RES) at the indicated concentration for 2 days. The magnification was 6000×.

Scanning electron microscopy investigation provided some new details on the structure of the AAPH-treated cells (Figure 2). Normal IMR-90 appeared as fully spread cells with typical fusiform shape. Most of the cells contained two tapering processes and exhibited few blebs. AAPH-treated cells displayed an enlarged and flattened morphology with numerous of blebs. At variance, scanning electron microscopy observation of IMR-90 samples treated with resveratrol or CR after AAPH administration clearly indicated that, although some blebs could be detected, the normal fusiform shape was generally maintained together with fully spread.

### Resveratrol and CR decreased apoptosis in AAPH-treated IMR-90 cells

**Figure 3:**
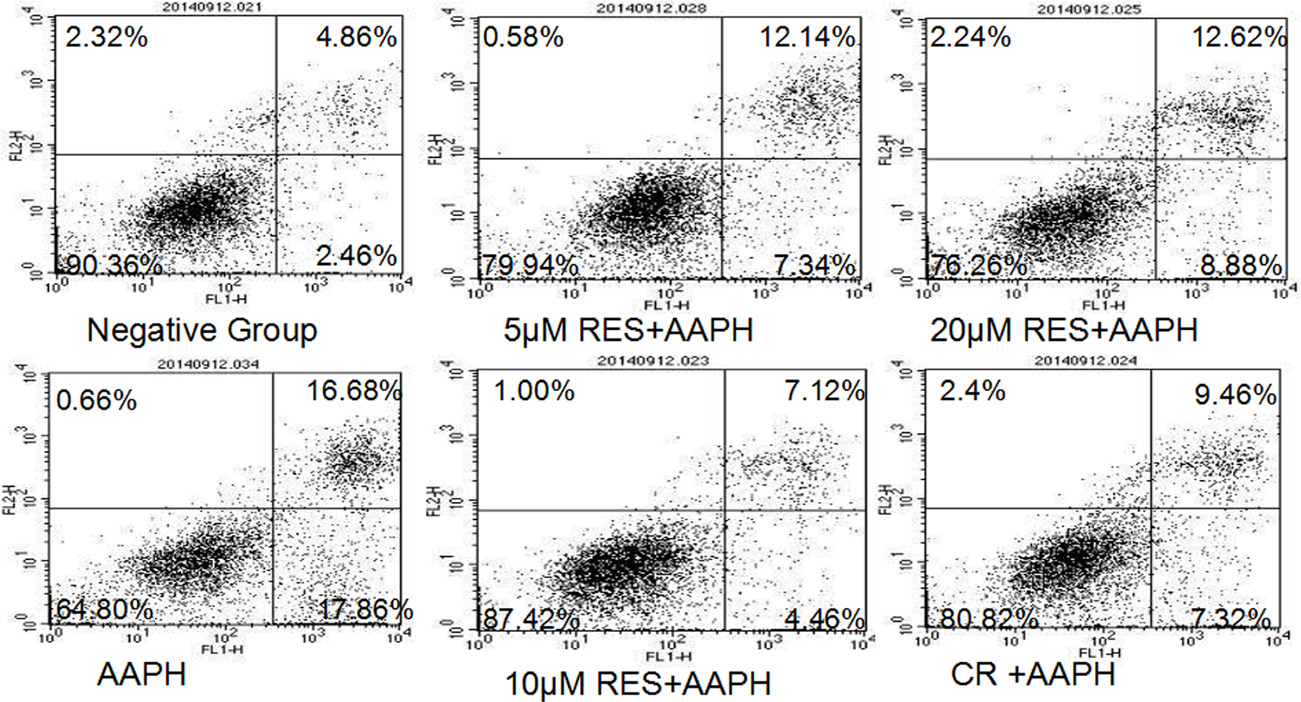
Resveratrol (RES) and caloric restriction (CR) decreased apoptosis in AAPH-treated IMR-90 cells Cells were stained with Annexin V-FITC/PI and analyzed by flow cytometry. The FL1 channel was used to detect annexin-V-FITC staining and the FL2 channel was for PI staining.

Phosphatidylserine on the surface of cells, considered as the hallmark of apoptosis in early phase, was determined by PI/Annexin V staining assay (Figure 3). The apoptotic cells compose of both early apoptotic cells stained with Annexin-V-FITC and late apoptotic or necrotic cells stained with Annexin-V-FITC and PI. The population of apoptotic cells was increasing significantly in AAPH group (34.54%), compared with negative group (7.32%). Whereas resveratrol (5 μM, 10 μM and 20 μM) or CR treatment was found to decrease apoptosis in AAPH-induced IMR-90 cells. In addition, 10 μM resveratrol treatment (11.58%) more efficiently decreased AAPH-induced apoptosis than CR treatment (16.78%).

### Change of general appearance and body weight

**Figure 4:**
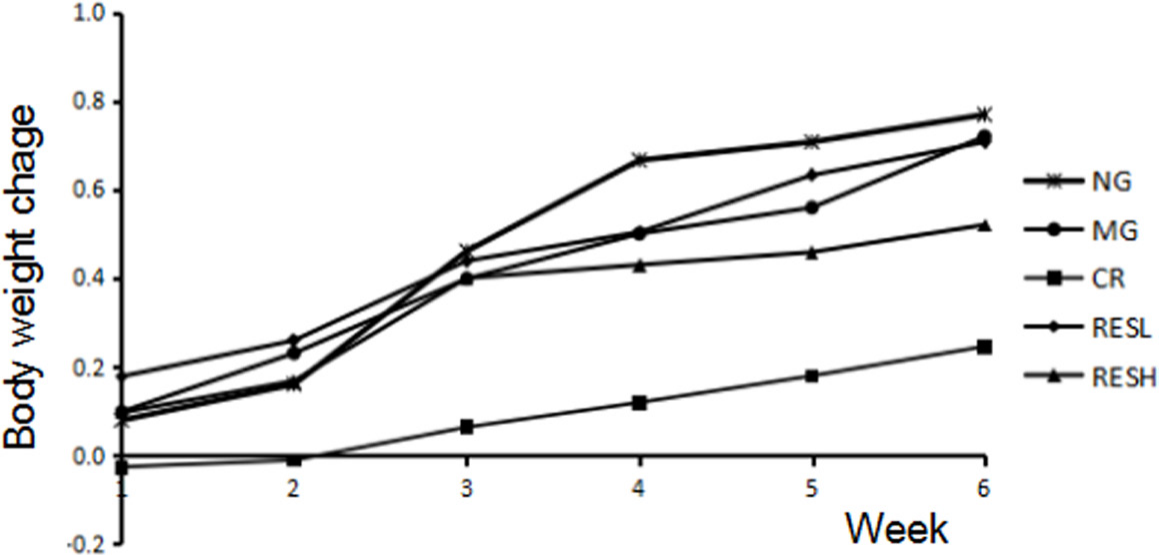
Effect of resveratrol and caloric restriction on the body weight of D-gal treated rats during the experiment All values were expressed as means ± SD. NG, negative control group; MG, model control group; RESL, low dose of resveratrol group; RESH, high dose of resveratrol group; CR, caloric restriction group.

During the entire experiment process, the aging model rats were received D-gal at dose of 200 mg/kg each day and general appearance was observed. Compared with that of normal control rats, the rats in D-gal model group gradually became apathetic, dull and slow in response; the hair of model group ones gradually lost elasticity and became brittle; the skin became thin, inelastic and sagged little by little. However, simultaneous treatment resveratrol or CR with D-gal in rats showed a reversal of aforementioned changes, as compared with the D-gal group. There was no difference in body weight between experimental groups within the period of treatment, except for CR group with almost no weight increase during the experiment (Figure 4).

### Resveratrol and CR improved the spatial learning and memory of SD rats

**Figure 5:**
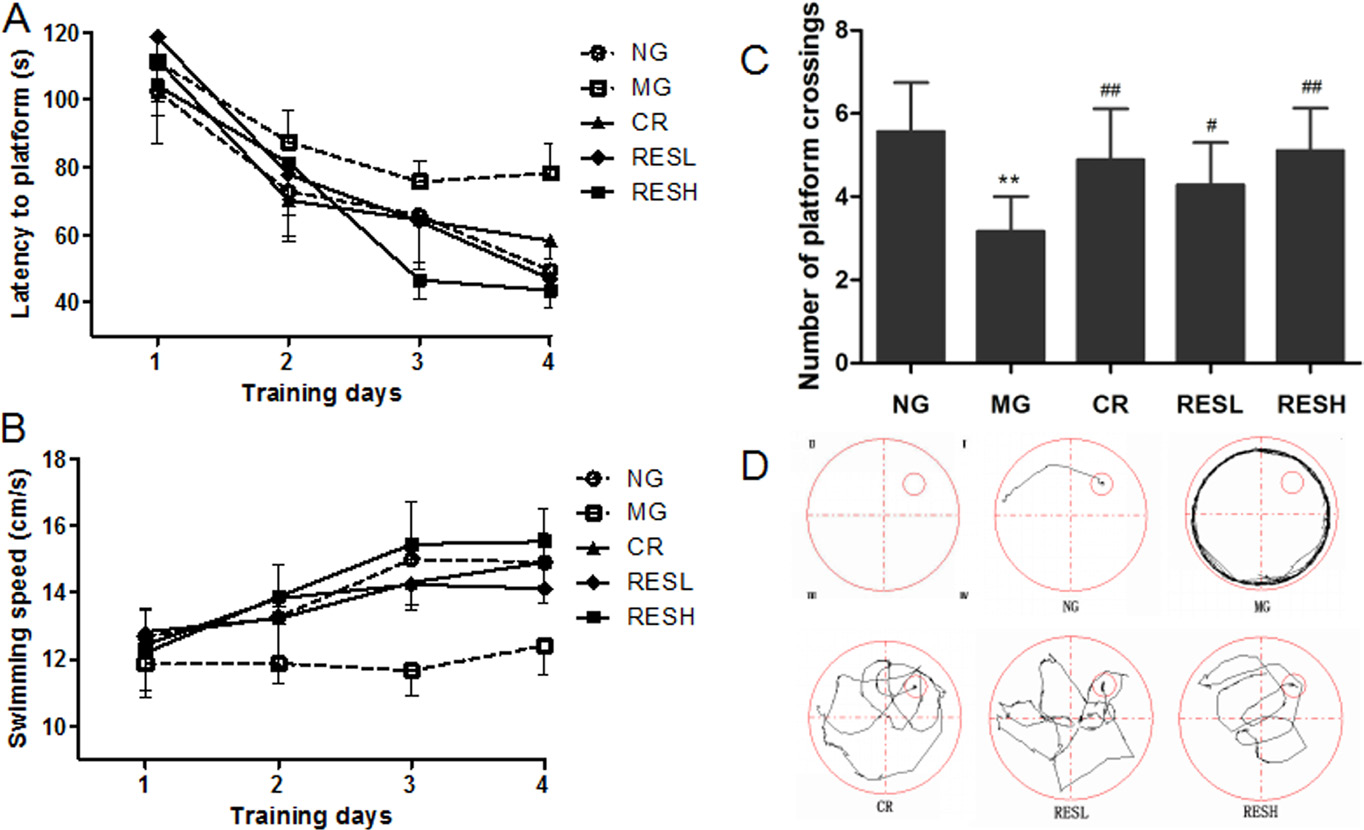
Effect of resveratrol and caloric restriction on the behavior of D-gal treated rats with Morris water maze (**A**) Comparison of latencies to platform during 4 training days. Each mouse was subjected to 4 trials per day. (**B**) Comparison of swimming speed during 4 training days. (**C**) Comparison of numbers of crossing over platform where the platform was removed for probe trial. (**D**) The typical swimming track in probe trial. All values were expressed as means ± SD. ^*^P < 0.05, ^**^P < 0.01 compared with negative control group; ^#^*P* < 0.05, ^##^*P* < 0.01 compared with model control group. NG, negative control group; MG, model control group; RESL, low dose of resveratrol group; RESH, high dose of resveratrol group; CR, caloric restriction group.

We assessed the spatial learning and memory ability of the rats by performing Morris water maze test. As shown in Figure 5, all five groups were able to learn the task successfully, as evidenced by gradually reduced escape latency and increased swimming speed in the 4 days of place navigation phase. Analyzed by the two-way ANOVA method, significant differences were shown both in mean escape latency and swimming speed between training days (F(3,138) = 41.660, *P* < 0.01; F(3,138) = 10.013, *P* < 0.01, respectively) and between treatments (F(4,46) = 36.762, *P* < 0.01; F(4,46) = 20.102, *P* < 0.01, respectively) but no interaction between the factors day and treatment (F(12,138) = 0.846, *P* > 0.05; F(12,138) = 1.084, *P* > 0.05, respectively). The escape latency in the D-gal group was markedly longer than that in the control group (*P* < 0.01) (Post-hoc analysis, Figure 5A). Meanwhile the rats in resveratrol + D-gal group or CR + D-gal group showed a reversal of aforementioned changes in escape latency compared with D-gal group (*P* < 0.01) (Figure 5A), and notably shortened the escape latency to the similar levels with control group. Similar results were obtained for the swim speed of rats (Figure 5B), and rats in resveratrol + D-gal group and CR + D-gal group displayed remarkable increases of the swim speed as compared with D-gal group (*P* < 0.01). However, rats in high dose of resveratrol + D-gal group displayed relatively longer escape latency and lower swim speed compared to the CR + D-gal group.

In the spatial probe phase (Figure 5C and 5D), it could be seen that there were remarkable reductions of platform crossings in the D-gal group compared to that in the control group (*P* < 0.01). Interestingly, the mean number of times the rats crossed the platform was significantly improved after administrating with resveratrol or CR, compared with that of the D-gal group (*P* < 0.01). However, rats in high dose of resveratrol + D-gal group crossed over the platform relatively much frequently than those in CR + D-gal group.

These results indicated that aging model rats had impairments in spatial learning and memory, while the treatment of resveratrol or CR could restore the age-related cognitive impairment caused by D-gal administration. Moreover, aging model rats treated with high dose of resveratrol showed relatively more effective on the behavior than treated with CR.

### Effect of resveratrol and CR on SOD, T-AOC, MDA and lipofuscin levels in aging rats

The SOD, T-AOC, MDA and lipofuscin levels in rats were evaluated to confirm whether the anti-aging effects of resveratrol and CR were mediated by alleviating the oxidative stress caused by D-gal administration. Table 1 showed the levels of SOD, T-AOC and MDA in serum, liver and brain in normal and aging rats. Exposure to D-gal significantly decreased the SOD and T-AOC levels but increased the MDA level in serum, liver and brain as compared with the control group (*P* < 0.05). Simultaneous treatment resveratrol or CR with D-gal in rats caused a decrease in the activity of MDA level but increased SOD and T-AOC levels as compared with the D-gal group. However, compared with CR treatment, the results showed that administration high dose of resveratrol was relatively superior.

**Table 1:** Antioxidants status in the tissue and blood of rats in each group

Index	Group	Liver	Brain	Serum
MDA(nmol/mg prot or nmol/ml)	NG	2.96 ± 0.26	2.93 ± 0.04	11.85 ± 0.44
	MG	3.40 ± 0.32^*^	3.51 ± 0.04^**^	14.12 ± 0.9^*^
	CR	3.04 ± 0.60^#^	3.05 ± 0.08^##^	12.82 ± 1.46
	RESL	3.18 ± 0.35	3.12 ± 0.07^*##^	11.97 ± 0.65
	RESH	2.88 ± 0.65^#^	2.83 ± 0.09^##∆^	9.41 ± 1.23^#^
SOD (U/mg prot or U/ml)	NG	307.38 ± 24.32	323.51 ± 33.17	85.50 ± 3.13
	MG	280.38 ± 18.10^*^	241.33 ± 46.06^**^	78.99 ± 2.33^*^
	CR	294.19 ± 24.21	305.03 ± 53.2^#^	81.81 ± 3.38
	RESL	272.65 ± 13.84	300.46 ± 46.59^#^	83.39 ± 2.56
	RESH	305.43 ± 28.61^#^	331.92 ± 52.45^##∆^	84.37 ± 2.77^#^
T-AOC (U/mg prot or U/mL)	NG	1.13 ± 0.05	0.71 ± 0.07	
	MG	0.97 ± 0.06^*^	0.57 ± 0.03^**^	
	CR	1.09 ± 0.04^#^	0.64 ± 0.04^#^	
	RESL	1.06 ± 0.08	0.70 ± 0.03^##^	
	RESH	1.19 ± 0.08^#∆^	0.89 ± 0.08^##∆^	

Note: Values were the mean ± SD.

^*^P < 0.05, ^**^P < 0.01 compared with control group (all groups);

^#^*P* < 0.05, ^##^*P* < 0.01 compared with model group (CR group and RES group);

^∆^*P* < 0.05, ^∆∆^*P* < 0.01 compared with CR group (RES group).

NG, negative control group; MG, model control group; RESL, low dose of resveratrol group; RESH, high dose of resveratrol group; CR, caloric restriction group.

**Figure 6:**
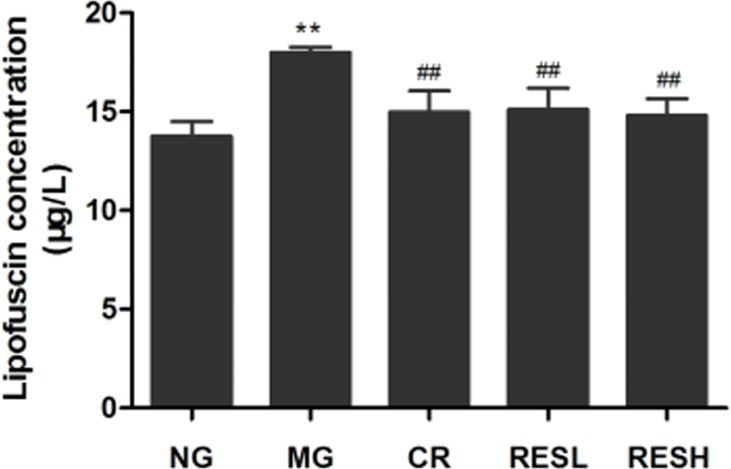
Effect of resveratrol and caloric restriction on lipofuscin level of aging rats All values were expressed as means ± SD. ^*^P < 0.05 compared with negative control group; ^#^ *P* < 0.05 compared with model control group. NG, negative control group; MG, model control group; RESL, low dose of resveratrol group; RESH, high dose of resveratrol group; CR, caloric restriction group.

Lipofuscin accumulation is one of the most consistent features of brain aging. As shown in Figure 6, D-gal-treated rats showed a significant increase of lipofuscin level compared with control group (*P* < 0.01). However, resveratrol and CR treatment decreased lipofuscin accumulation in cerebral cortex of aging rats in comparison to D-gal treated rats (*P* < 0.01). Moreover, there were no differences between resveratrol + D-gal group and CR + D-gal group (*P* > 0.05).

### Resveratrol and CR up-regulated telomerase activity in aging rats

**Figure 7:**
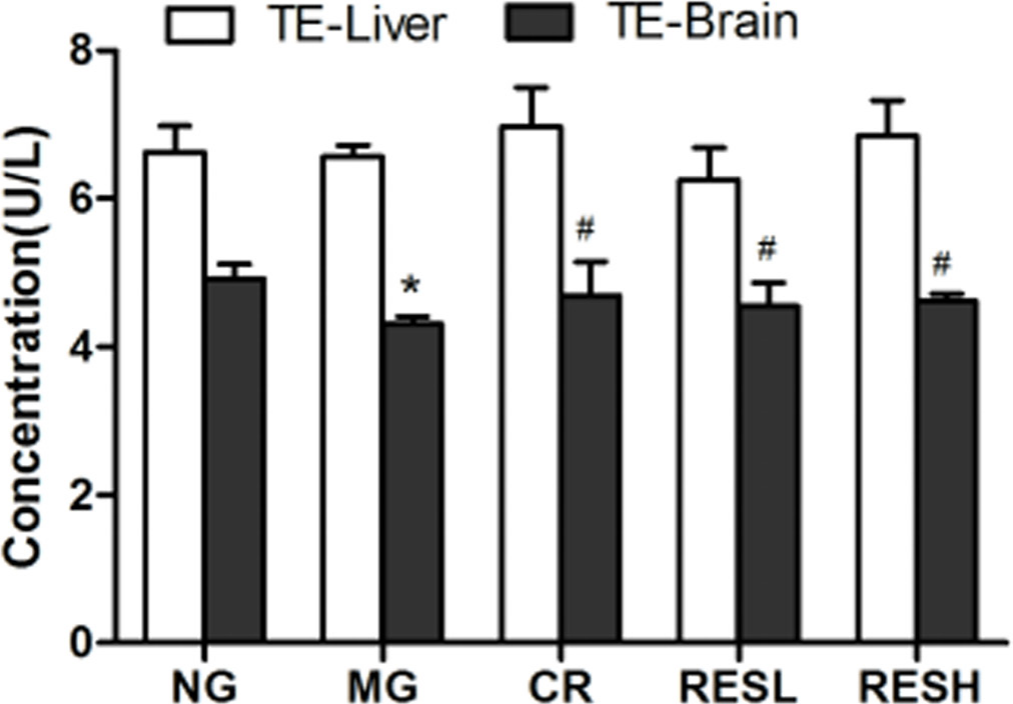
Effect of resveratrol and caloric restriction on the TE activity in aging rats All values were expressed as means ± SD. ^*^P < 0.05 compared with control group; ^#^*P* < 0.05 compared with model group. NG, negative control group; MG, model control group; RESL, low dose of resveratrol group; RESH, high dose of resveratrol group; CR, caloric restriction group; TE, telomerase.

We evaluated the effect of resveratrol and CR on telomerase activity in liver and brain tissues (Figure 7). Our results showed that treatment had no significant effect on the telomerase activity in liver tissues (*P* > 0.05). However, the telomerase activity was reduced in the D-gal administration group compared with the control (*P* < 0.05). Meanwhile, resveratrol or CR could up-regulate telomerase activity in the D-gal administration plus resveratrol or CR treatment group. Moreover, there were no differences between the resveratrol group and the CR group (*P* > 0.05).

### Resveratrol and CR increased AAPH-induced SIRT1 mRNA expression

**Figure 8:**
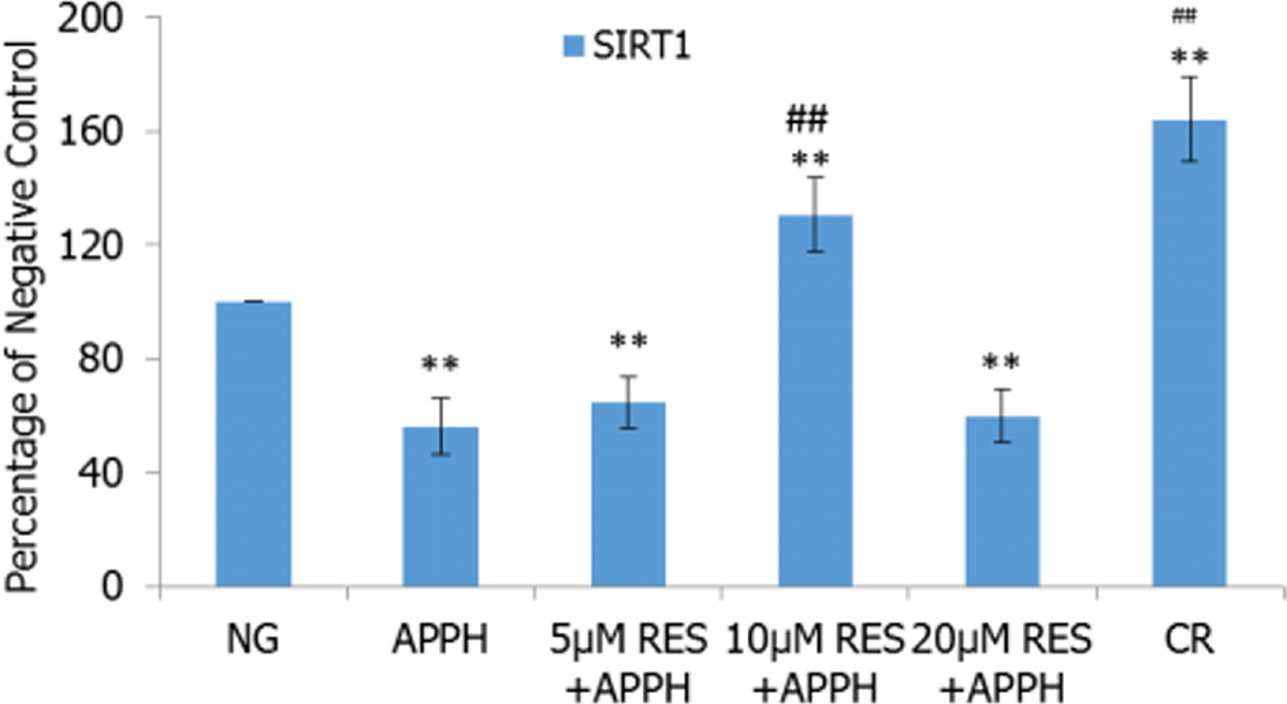
Effect of resveratrol (RES) and caloric restriction (CR) on SIRT1 mRNA expression in IMR-90 cells All values were expressed as means ± SD. ^**^P < 0.01 compared with negative control group; ^##^ *P* < 0.01 compared with model control group.

To explore the role of SIRT1 in the treatment effects of resveratrol and CR, the expression levels of SIRT1 mRNA in IMR-90 cells and tissues were examined by quantitative real-time RT-PCR. The results demonstrated that the expression of SIRT1 mRNA in IMR-90 cells was significantly reduced by AAPH. Resveratrol and CR could efficiently recover SIRT1 mRNA expression levels in AAPH-treated cells, and 10 μM resveratrol was the optimal concentration to stimulate the expression of SIRT1 mRNA in IMR-90 cells (*P* < 0.01; Figure 8)

### Effect of resveratrol and CR on the mRNA expressions of SIRT1, p53 and Foxo3a in tissues of aging rats

**Figure 9:**
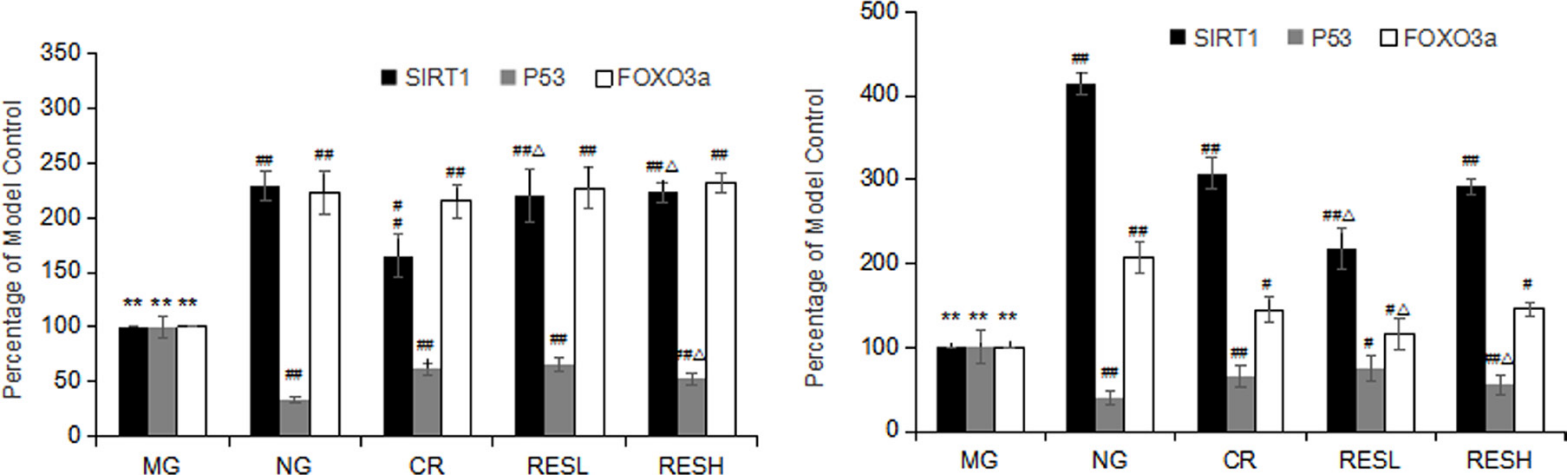
(**A**) Effect of resveratrol and caloric restriction on mRNA expressions of SIRT1, p53 and Foxo3a in brains of aging rats. (**B**) Effect of resveratrol and caloric restriction on mRNA expressions of SIRT1, p53 and Foxo3a in livers of aging rats. All values were expressed as means ± SD. ^*^P < 0.05, ^**^P < 0.01 compared with negative control group; ^#^*P* < 0.05, ^##^*P* < 0.01 compared with model control group; ^#^*P*<0.05, ^##^*P*<0.01 compared with CR group (RES group). NG, negative control group; MG, model control group; RESL, low dose of resveratrol group; RESH, high dose of resveratrol group; CR, caloric restriction group.

Oxidative stress conditions often induce SIRT1 expression and activity. SIRT1 modulates the functions of key survival factors including p53 and Foxo3a [9]. To determine the effect of resveratrol and CR on the mRNA levels of SIRT1, p53 and Foxo3a in D-Gal induced aging rats, quantitative RT-PCR assays were performed. Compared to the corresponding negative control group, the levels of SIRT1 and Foxo3a mRNA expression were significantly decreased, but p53 level was significantly increased in D-Gal group (*P* < 0.01; Figure 9A and 9B). Furthermore, simultaneous treatment resveratrol or CR with D-gal in rats caused an increase in the levels of SIRT1 and Foxo3a mRNA expressions but decreased p53 levels as compared with the model control group (*P* < 0.05). However, compared with CR treatment, the level of SIRT1 mRNA expression was significantly increased in livers of high dose of resveratrol + D-gal group, the level of p53 mRNA expression was significantly decreased in livers and brains of high dose of resveratrol + D-gal group (*P* < 0.05).

### Effect of resveratrol and CR on the expressions of main SIRT1-associated protein in brain tissues of aging rats

**Figure 10:**
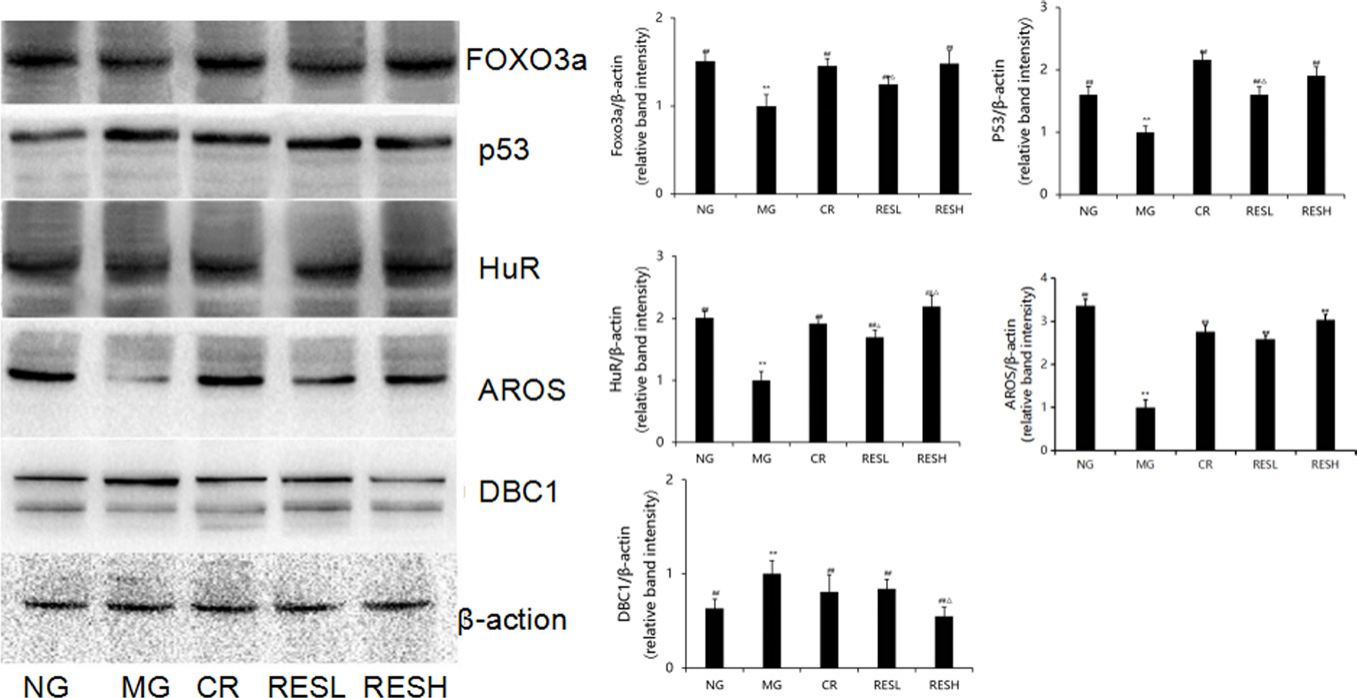
Effect of resveratrol and caloric restriction on protein expressions of p53, FOXO3a, HuR, AROS and DBC1 in brains of aging rats All values were expressed as means ± SD. ^*^P < 0.05, ^**^P < 0.01 compared with negative control group; ^#^*P* < 0.05, ^##^*P* < 0.01 compared with model negative group; ^∆^*P* < 0.05 compared with CR group (RES group). NG, negative control group; MG, model control group; RESL, low dose of resveratrol group; RESH, high dose of resveratrol group; CR, caloric restriction group.

FOXO3a and p53, which are regulated by SIRT1, are the downstream molecules of SIRT1. Simultaneously, SIRT1 activity is regulated by its upstream molecules, for instance, DBC1 (also known as KIAA1967), AROS (also known as RPS19BP1) and the tumour suppressor HuR (also known as ELAVL1) [9, 10]. To determine the effect of resveratrol and CR on the protein levels of p53, FOXO3a, HuR, AROS and DBC1 in D-Gal induced aging rats, western blot assays were performed. Compared to the corresponding negative control group, the levels of FOXO3a, AROS and HuR protein expressions were significantly decreased, but p53 and DBC1 levels were significantly increased in D-gal group (*P* < 0.01; Figure 10). Furthermore, simultaneous treatment resveratrol or CR with D-gal in rats caused an increase in protein expressions of FOXO3a, AROS and HuR but decreased p53 and DBC1 levels as compared with the model control group (*P* < 0.05). However, compared with CR treatment, the level of HuR protein expression was significantly increased but DBC1 level was significantly decreased in brains of high dose of resveratrol + D-gal group (*P* < 0.05). There were no differences between resveratrol + D-gal group and CR + D-gal group in levels of p53, FOXO3a and AROS protein expression (*P* > 0.05).

### FOXO3a and p53 expressions in brain and liver tissues

**Figure 11:**
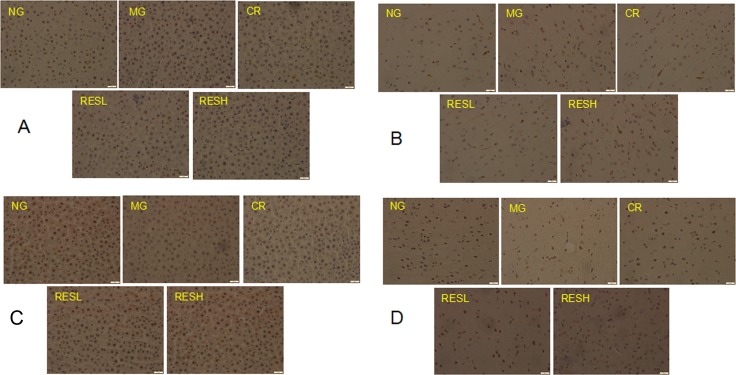
Immunohistochemical analysis of p53 (A, B) and FOXO3a (C, D) expressions in liver (A, C) and brain (B, D) tissues of aging rats The magnification was 20×. NG, negative control group; MG, model control group; RESL, low dose of resveratrol group; RESH, high dose of resveratrol group; CR, caloric restriction group.

The expression status of FOXO3a and p53 were determined in brain and liver tissues by immunohistochemistry. Immunohistochemical analysis of FOXO3a and p53 revealed that FOXO3a expression in brain and liver tissues was significantly decreased, and p53 expression was significantly increased in D-Gal group, compared with negative control group (*P* < 0.05, Figure 11). Moreover, simultaneous treatment resveratrol or CR with D-gal in rats caused an increase in FOXO3a expression but decreased p53 expression as compared with the model control group (*P* < 0.05). However, there were no differences between resveratrol + D-gal group and CR + D-gal group in p53 and FOXO3a expressions (*P* > 0.05).

## DISCUSSION

The basic chemical process underlying aging was first advanced by the free radical theory of aging [11]，and oxidative stress is believed to be a primary factor in the normal process of aging. Free radical initiator AAPH was used to induce oxidative stress and establish a model of oxidative stress-induced cellular senescence in human IMR-90 cells [12, 13]. The advantages of this method are that the AAPH decomposes thermally to generate radicals without biotransformations or enzymes, and the rate of radical generation could be easily controlled by adjusting the concentration of the initiator [12]. AAPH was found to inhibit the proliferation of human IMR-90 cells in a concentration and time dependent manner. Results showed that 2 d incubation with AAPH (1 mM) significantly increased the percentage of SA β-Gal positive ratio, the population of apoptotic cells, reduced the mRNA expression of SIRT1, and induced an enlarged and flattened morphology. These indicated that oxidative stress resulted by AAPH led IMR-90 cells to senescence. Based on this cellular senescence model, 10 μM resveratrol treatment more efficiently inhibited AAPH-induced senescence and apoptosis than CR treatment.

D-gal animal model is an internationally recognized aging animal model and has been widely used in the field of anti-aging medicines. D-gal is a physiological nutrient that is normally metabolized by D-galactokinase and galactose-1-phosphate in animals. However, over-supply of D-gal, which is not metabolized by above enzymes but accumulate in the cells, leads to oxidative stress and cellular damage [14]. Thus, long-term administration of D-gal induces changes that resemble natural aging in animals, such as a shortened lifespan, cognitive dysfunction, neurodegeneration, oxidative stress, decreased immune responses, and advanced glycation endproduct (AGE) formation [15]. The present study indicated the successful establishment of the mimetic aging model, evidenced by remarkable learning and memory impairment, MDA production, lipofuscin accumulation, decline in T-AOC and SOD, and down-regulation of telomerase activity in brain. Meanwhile, resveratrol or CR treatments protected the D-gal induced rats against oxidative stress by elevating the activity of antioxidant enzymes, decreasing the levels of lipid peroxidation products, and keeping the balance of oxidative and antioxidative systems. In behavioral and telomerase activity tests, the treatment of resveratrol or CR could reverse D-gal-induced cognitive impairment and telomerase activity. Moreover, aging model rats treated with high dose of resveratrol showed relatively more significant effect on the the behavior than treated with CR.

Studies have demonstrated that CR, a reduction of 10–40% in intake of a nutritious diet, could retard aging and increase lifespan, thus one commonly used level of CR (40% reduction in food intake) was applied in CR group rats in present study. Nevertheless, some investigations revealed that no beneficial effect of CR on longevity in naturally aged primates [16]. Similarly, although resveratrol treatment was shown to induce CR-like effects on energy metabolism and metabolic profile in obese humans [17], it did not improve metabolic function in non-obese women with normal glucose tolerance [18]. The reason for the differences between these studies is not clear but it might be that CR and resveratrol work to restore homeostasis in metabolically compromised individuals, and less so in healthy individuals, a possibility consistent with known functions of SIRT1 in animal studies [19]. Therefore, D-gal induced aging rat model were applied for comparing anti-aging activities of resveratrol or CR , rather than naturally aged rat model.

SIRT1, an (NAD+)-dependent deacetylase, has gained much attention because of its multiple roles in life span extension, stress resistance and apoptosis reduction [20, 21]. Accumulating studies have strongly implied that SIRT1 was a key target of resveratrol and CR [22]. There are vast numbers of downstream molecules of SIRT1, including p53, Foxo1, Foxo3, Foxo4 and E2F1, which are regulated by SIRT1. Simultaneously, SIRT1 activity is regulated by its upstream molecules, for instance, p53, HIC1, E2F1, DBC1, HuR and AROS. Acute nutrient withdrawal activates a transcriptional program at the SIRT1 promoter that is directed by the transcription factors Foxo3a and p53. As p53 represses SIRT1 gene expression, its removal by Foxo3a activates SIRT1 transcription [23]. DBC1 and AROS were recently described as direct negative and positive regulators of SIRT1 activity, respectively, HuR shows the effect of reducing levels of SIRT1 expression in aged senescent cells [9, 10].

CR retards aging and extends median and maximal life span in a variety of species, including rats, mice, fish, flies, worms, and yeast. However, such a rigorous dietary programs in free living persons has raised ethical and methodologic issues [24]. At present, increasing health span might be more important than simply prolonging lifespan. Recent investigations suggest that resveratrol is one of the most potent instigators of SIR2 activity among all the plant polyphenols [25], and it influences longevity through similar mechanisms as seen in calorie restriction. This results showed that CR and resveratrol had similar activities of recovering SIRT1 mRNA expression levels, increasing the protein expressions of FOXO3a, AROS and HuR and decreasing p53 and DBC1 levels. Meanwhile, multiple lines of compelling evidence indicate the beneficial effects of resveratrol on neurological, hepatic, and cardiovascular systems.

## MATERIALS AND METHODS

### Cell culture, stress and treatments

The human diploid fibroblast strain IMR-90 was obtained from ATCC. Cells were grown in minimum essential medium (MEM) (Gibco, UK) supplemented with 10% fetal bovine serum (FBS) (Gibco) at 37°C in 5% CO_2_. After confluence had been reached, the cells were seeded into 6-well culture plates. One day later, cells were treated for 48 h with 1 mM 2,2′-azobis (2-amidinopropane) dihydrochloride (AAPH) (Sigma, USA) diluted in MEM + 10% FBS. A 48 h incubation with free radical initiator AAPH establishes a model of oxidative stress-induced cellular senescence [12, 13]. Controls cells were incubated in culture medium alone. After AAPH treatment, IMR-90 were washed with cold phosphate buffer saline (PBS) pH 7.4 and incubated with fresh culture medium or culture medium containing resveratrol (resveratrol group) or MEM supplemented with 3% FBS (CR group) for an additional 48 h before harvest.

### SA β-gal staining activity

The appearance of biomarkers of senescence was checked (decrease of the proliferative potential and increase in SA β-gal-staining) [26] from the day after treatment. Then the cells were stained following the manufacturers’ instruction of the SA β-gal Staining Kit (CST, USA). After staining, at least 300 cells in several fields were examined and SA β-gal positive cells were counted. These experiments were repeated three times, and the results were presented as the mean values with standard deviations (SD). To avoid a nonspecific staining possibly due to cell confluency, SA β-gal histochemical staining was performed with subconfluent cells.

### Scanning electron microscopy analysis

Cells were grown on coverslips in 6-well culture plates as described above. After resveratrol and CR treated, coverslips were removed from the plates, then the cells fixed in 2.5% phosphate-buffered glutaraldehyde, and post-fixed in 1% buffered osmium tetroxide. Fixed specimens were immersed in t-butyl alcohol after dehydration through a graded series of ethanol. The specimens were freeze-dried from t-butyl alcohol, then evaporative-coated with gold using auto fine coater (JFC-1600, JEOC, Japan), and examined using scanning electron microscope (JEOL, JSM-6510LV, Japan).

### Apoptotic analysis

After resveratrol or CR incubation, all cells were harvested with trypsin and washed twice with PBS, followed by resuspended in 400 μL Annexin V binding buffer. Then the cells were stained following the manufacturers’ instruction of the Annexin V-FITC cell apoptosis detection kit (BestBio, China). A FACSCalibur flow cytometer (Becton-Dickinson, San Jose, CA) was used to detecte fluorescence, and the percentage of apoptotic cells was calculated by the internal software system of the FACSCalibur. Approximately 10^4^ cells were analyzed for each trail.

### Animal procedures

Male albino Wistar rats, weighing approximately 200 ± 10 g, were obtained from the Experimental Animal Center of Tongji Medical College, Huazhong University of Science and Technology (SCXK 2010-0009). The animals were acclimated for 2 weeks before dosing in Experimental Animal Center of Hubei University of Chinese Medicine, during which time they had free access to food and water ad libitum. After acclimation, 50 rats were selected and divided into five groups of ten each. Animals were maintained per the national guidelines and protocols approved by the Institutional Animal Ethical Committee (IAEC).

Group I (Negative control group) rats received the vehicle alone (1 mL/kg bodyweight of 0.9% of saline) for 6 weeks. Group II (Model control group) rats received D-gal (200 mg/kg bodyweight, Sigma Chemical, St. Louis, MO) for 6 weeks. Group III (Low dose of resveratrol group) rats received D-gal and resveratrol (50 mg/kg bodyweight) dissolved in 5% dimethyl sulfoxide (DMSO) for 6 weeks. Group IV (High dose of resveratrol group) rats received D-gal and resveratrol (100 mg/kg bodyweight). Group V (CR group) rats received D-gal and CR (fed a diet that was 40% lower in calories than the ad libitum fed rats [27]) for 6 weeks. D-gal were given intraperitoneally, whereas resveratrol were given orally for the entire duration of the experiment. The rats were sacrificed on the last day of treatment, then the blood, sera and organs were immediately collected for bioassay or stored at −80°C for later use.

### Behavioural tests

The Morris water maze test was designed to assess the rats’ capacity of the spatial learning and memory after 6-week post-injury [28]. The Morris water maze test consisted of 4-day learning and memory training and a probe trial on day 5. Animals were trained in a circular pool (155 cm in diameter) with visual cues. An escape platform (9.0 cm in diameter) was submerged 1.0 cm below the surface of the pool water, which was maintained at 25 ± 2°C. The location of the platform remained in the center of northwest quadrant throughout the 4-day training period. On each day, rats were trained for one morning block and one afternoon block. The rat swam freely until it found the platform within 120 s. If failed, it was placed on the platform for 10 s. The escape latency (finding the submerged escape platform) and the swim speed were recorded. The probe trial was made by removing the platform and allowing each rat to swim freely for 120 s inside the pool. The number of the platform crossings in the trained quadrant (where the platform was removed) were recorded with a computerized video system. The visible platform version of water maze was not performed after significant difference in swim speed was observed between rats treated with D-gal and vehicle.

### Detection of oxidative stress-associated biological indicators

The levels of MDA, SOD, and T-AOC in blood and tissues were determined photometrically in accordance with the manufacturer’s protocol by using commercially available enzymatic assay kits (Nanjing Jiancheng Bioengineering Institute, Nanjing City, P. R. China). All the experiments described in this section were performed in triplicate to obtain means and SD.

Lipofuscin level was determined by the Sohal method [29]. In brief, we weighed 200 mg cerebral cortex, added 2 ml chloroform-methanol (2:1) extract, homogenized and filtered, then washed the residue with the extract, combined the filtrates, added the extract to 5 ml and measured the fluorescence intensity on a fluorescence spectrometer. Emission wavelength was 435 nm and excitation wavelength was 365 nm. The spectrofluorometer was standardized to give a deflection of 50 at the above wavelengths with a 1 μg/ml solution of quinine bisulfate in 0.1 M H_2_SO_4_. The results were expressed as relative fluorescent units/ml chloroform/g wet tissue weight.

### Detection of TE activity

For telomerase extraction approximately 30 mg of tissue was washed twice in ice-cold PBS, and finally homogenized in about 150 ml of PBS. TE activity was measured using a TE ELISA kits according to the manufacturer’s instructions (Nanjing Sen Beijia Biological Technology Co., Ltd., Nanjing, China). The limits of sensitivity of the assays were 0.8 IU/L for TE.

### RT-PCR analysis

The mRNA expression levels of SIRT1, Foxo3 and p53 in liver and brain tissues were analyzed using RT-PCR analysis. The total RNA was extracted using Trizol, their quantity and purity were assessed by a ultra micro spectrophotometer (Thermo Fisher Scientific, USA) based on the absorbance measurement at 260 and 280 nm. After quantification, cDNA was synthesized using a FastQuant RT kit (Tiangen Biotech, Beijing, China) according to the manufacturer’s instructions. The levels of SIRT1, Foxo3, p53 mRNA expression were measured by RT-PCR using TIANGEN SuperReal PreMix Plus (Tiangen Biotech, Beijing, China) with specific primers (Table 2). Quantitative real-time PCR was performed with an LightCycler 480°C PCR system (Roche, Basel, Switzerland) using 20 μL as the total volume of each reaction. PCR amplification was initiated by 15 min of denaturation at 95°C, and then followed by 40 cycles of 95°C for 10 s, 59–63°C (annealing temperature) for 20 s and 72°C for 30 s, and a final incubation at 72°C for 5 min. The obtained cycle threshold number of each gene (Ct value) was normalized into fold of relative changes according to the equation of 2-∆∆CT method [30].

**Table 2:** Primer list

Gene	Forward Primer (5′ to 3′)	Reverse Primer (5′ to 3′)
SIRT1	CTTCAGGTCAAGGGATGGTAT	GCGTGTCTATGTTCTGGGTAT
Foxo3	TCCTTTAACAGTACCGTGTTCGGA	GCAGGTCTTGGAGTGTCTGGTTG
p53	GCTCCGACTATACCACTATCCACTAC	CAGGACAGGCACAAACACGA
h β-actin	TGACGTGGACATCCGCAAA	CTGGAAGGTGGACAGCGAGG
rat β-actin	CACCCGCGAGTACAACCTTC	CCCATACCCACCATCACACC
rat GAPDH	ACAGCAACAGGGTGGTGGAC	TTTGAGGGTGCAGCGAACTT

### Western blot analysis

The protein expression levels of p53, FOXO3a, HuR, RPS19BP1, DBC1 in brain tissues were analyzed using western blotting analysis. The total protein of brain tissues was extracted using RIPA Lysis Buffer (Beyotime, China) according to the manufacturer’s instructions. Protein concentrations were determined using the BCA Protein Assay Kit (Beyotime, China). 5 mg of proteins were separated using 12% SDS-polyacrylamide gels and transferred onto a PVDF membrane (0.45 μm, Millipore, USA). The blots were blocked with 5% non-fat milk in TBS, then incubated overnight at 4°C with the appropriate dilution of primary antibodies: anti-p53 (SC-6243, Santa Cruz Biotechnology), anti-FOXO3a (#12829, Cell Signaling Technology), anti-HuR (#12582, Cell Signaling Technology), anti-AROS (Ab201091, abcam), anti-DBC1 (#5857, Cell Signaling Technology), anti-β-action (AC004, ABclonal Technology). After washing the membranes to remove excess primary antibody, the membranes were incubated for 1 h at room temperature with the appropriate secondary antibodies at a dilution of 1:2000-1:5000 (AC004 or AS003, ABclonal Tehcnology). The membranes were washed 3 times and then visualized using Pierce ECL Western Blotting Substrate (Thermo Scientific). β-action was used as an internal control.

### Immunohistochemistry

The tissue samples were fixed in 10% neutral-buffered formalin within 1 h after surgical removal and paraffin-embedded using standard procedures. Then the fixed paraffin-embedded specimens were cut into 5-μm-thick sections, which were deparaffinized in xylene, rehydrated in ethanol, and microwave treated for antigen retrieval. The appropriately diluted primary antibody, anti-p53 (SC-6243, Santa Cruz Biotechnology) and anti-FOXO3a (#12829, Cell Signaling Technology), were incubated for 60 min at room temperature in a humidity chamber. Slides were then rinsed in PBS and subsequently incubated with the secondary antibody against anti-rabbit antibody and the visualization of HRP (concentration 1:300, #074-1506, KPL). Then the slides were washed and the sections were developed in the enzyme substrate diaminobenzidine (DAB, Sigma) solution. Images of immunohistochemically stained sections were captured by the Olympus digital microscope (IX-73P1F, Olympus, Japan).

### Statistical analysis

Results were expressed as means ± SD for at least three independent experiments and were analyzed using the SPSS 18.0 software. Group differences in the escape latency and swim speed in the Morris water maze training task were analyzed using two-way analysis of variance (ANOVA) with repeated measures, the factors being treatment and training day. The other data were analyzed with one-way ANOVA followed by post-hoc Tukey test. The value of *P* less than 0.05 were considered significant.

## CONCLUSIONS

Resveratrol and CR exhibited similar anti-aging activities both *in vitro* and *in vivo*, evidenced by their ability to inhibit AAPH-induced senescence and apoptosis, restore the age-related cognitive impairment caused by D-gal administration. Their anti-aging mechanisms included up-regulating TE activity, decreasing oxidative damage, regulating SIRT1 pathway. Overall, 10 μM resveratrol *in vitro* and high dose of resveratrol *in vivo* exhibited relatively stronger activities of anti-aging and stimulating SIRT1 level. These results implicated the potential of resveratrol as a CR mimetic.

## Abbreviations

AAPH: 2,2′-azobis (2-amidinopropane) dihydrochloride
AMPK: adenosine monophosphate activated protein kinase
ANOVA: analysis of variance
AROS: active regulator of SIRT1
CR: caloric restriction
DBC1: deleted in breast cancer 1
D-gal: D-galactose
FBS: fetal bovine serum
Foxo3a: forkhead box 3a
HuR: Hu antigen R
MEM: minimum essential medium
PBS: phosphate buffer saline
PGC-1α: peroxisome proliferator activated receptor G coactivator-1α
RES: resveratrol
SA β-gal: senescence-associated β-galactosidase
SD: standard deviations
SIRT1: silencing information regulator
TE: telomerase
